# Sensor based precise nitrogen application augmented productivity and profitability of mustard (*Brassica juncea* L.)

**DOI:** 10.1371/journal.pone.0304206

**Published:** 2024-06-21

**Authors:** Vasudev Meena, Mohan Lal Dotaniya, Murli Dhar Meena, Ram Swaroop Jat, Mukesh Kumar Meena, Ram Lal Choudhary, Hari Singh Meena, Bheeru Lal Meena, Pramod Kumar Rai

**Affiliations:** ICAR-Directorate of Rapeseed-Mustard Research, Bharatpur, India; IISS: Indian Institute of Soil Science, INDIA

## Abstract

Unremitting decline in crop productivity and nutrient recovery are resulted due to dearth of need based fertilizer recommendation over blanket application apart from nitrogen pollution in several means. An advance nutrient management tactic, GreenSeeker (GS) has developed and used in many field crops following the principle of four “R” (right source, right amount at right time, and place) nutrients stewardship technologies. But no studies have been conducted for evaluation of GS in mustard for improving productivity, profitability and nutrient use efficiency (NUE) while minimizing environmental risks. With this objective, a study was planned to conduct an experiment in *rabi* season of 2021–22 and 2022–23 to assess optical sensor based nitrogen management in mustard over blanket recommendation. The experiment was comprised of ten N treatments including control in randomized block design in triplicates. Research findings indicated that application of GreenSeeker based N significantly improved all growth traits and yield parameters in *Brassica juncea* L. Per cent enhancement in seed yield, net monetary returns and benefit-cost ratio was higher as 19.3 and 64.5%, 125.1 & 36.2% and 58.8 & 24.4%, respectively under GS based multi split N application over RDF and control. Further, real time N management with GS acquired higher crop production efficiency (CPE) (19.9 kg/day) with lesser cost/kg production (Rs 15.7/kg). Split application of N using GS increased oil yield by 79.9 and 26% over control and recommended dose of fertilizer (RDF) with maximum oil content (42.3%), and increases soil organic carbon (SOC) content by 16.1% from its initial value. Moreover, GS crop sensor could be the probable solution to minimize the crop nitrogen requirement by 15–20% with a yield enhancement of about 18.7% over RDF.

## Introduction

Declining factor productivity, nutrient recovery due to indiscriminate and rein less use of fertilizers are the major and serious constraints for nitrogenous (N) fertilizers mainly, which dealt with the sustainability of cropping systems [1]. Efficient nitrogen fertilizer management is getting affected due to wider temporal and land variability with respect to soil N supply when broadcasting blanket fertilizers. Also, an ignorant farmer uses higher quantities of nitrogen fertilizers than what recommended for achieving better economic harvest. In current agricultural system, improving NUE have been most critical and daunting research issues [2]. Accelerating use efficiency of inputs (like nutrients, fertilizers, seeds) by optimizing their demand with supplies from various chemical and non-chemical (organic and inorganic) sources in an effective manner are the need for minimizing over uses of fertilizers to sustain production and to enhance profitability and NUE. Hence, nutrient management approaches should be more responsive to temporal variations with respect to plant nutrient requirement to accomplish supply–demand synchrony and if it is not harmonized with plant needs, nutrients wastage occurred via soil–plant system would be more, causing low fertilizer use efficiency.

Enhanced harmonization among the nitrogen need and supply by various available sources (soil, chemical fertilizers or manures) during crop growth period on site-specific basis can be conducive in boosting input use effectiveness, optimizing fertilizer nitrogen use [3] and reduces the potential for environmental contamination [4]. Studies reported that uptake of nitrogen top-dressed at panicle emergence was up to 78 percent. The SSNM is an optional way to imbalance and indiscriminate blanket fertilizer recommendations that supply soil N to the plants with its demand to match synchronization over time and space by following right quantity of fertilizer nitrogen at right time all through crop growing period and minimized wastages of nitrogen from soil-plant system as less as possible. Real time management of nitrogen fertilizers with their optimization (use) play crucial role in minimizing the environmental losses of N besides increasing nutrient uptake [5]. In this direction, splitting of nitrogenous fertilizers application is important key strategy employed to upsurge crop productivity and nutrient recovery [6] by adjusting N application time coinciding with crop N requirement while meantime abating the hazards of N losses [7, 8]. Under such conditions, use of diagnostic tools (LCC, SPAD meter, GreenSeeker) may help to assess real-time N necessity of mustard during crop growing period in the field condition and to optimize excesses usage of fertilizer N, reducing their losses due to various means besides abating peril of environmental degradation. Optical sensor based N management is gaining much popularity and in recent days, its use increased widely in agriculture to detect N stress by measuring red and near-infrared (NIR) spectral responses from plant canopies [9–11]. Bijay Singh et al. [12] reported that sensor-guided N management in rice resulted in equivalent yield as blanket fertilizer recommendation in farmer practices, however, significant improvement was observed in recovery efficiency (by 5.5–21.7%) and agronomic efficiency (by 4.7–11.7 kg grain/kg N applied) with reduced N rates. Li et al. [13] and Bijay-Singh et al. [14] showed that prediction of wheat response to N applications guided through an optical sensor was positively correlated to measured N response and resulted in augmented NUE. So far, the use of GreenSeeker for real time N application is focused in cereal based crops like rice, maize, wheat etc. The study on GS guided N management in mustard crop was not attempted up to now.

Hence, the author/s has made endeavour for effective management of N fertilizer using GreenSeeker optical sensor to ensure optimum yield and seed quality of *Brassica* species. Therefore, a study was designed to test the hypothesis that real-time N delivery at appropriate rates may significantly influence *Brassica juncea* L. growth and development, productivity, and oil yield, as well as speed up the acquisition of more nutrients in mustard-based cropping systems.

## Methodology

### Experimental site and location

A field experiment was executed at Agronomy farm section, ICAR-DRMR, Bharatpur (27°12’8.9" N latitude and 77°27’18.8" E longitude) at an altitude of 178.4m above MSL) in *rabi* season of 2021–22 and 2022–23 with Indian mustard (*Brassica juncea* L.) in sub-tropical, semi-arid climate. The average annual precipitation of the area is approximately 650 mm, and 85% of that received during the months of July-August by south-west monsoon. The daily average high and low temperature (15–34.8 and 4–21.8°C), relative humidity (70.5–97.3 and 45.3–89.4%,), bright sunshine hours (0–10 hours/day) and 0–7.6 km hr^-1^ of wind velocity, respectively, were recorded for the period of crop growth. The soil was clay loam in texture with 0.41% organic carbon, 225.1 kg/ha KMnO_4_ oxidizable nitrogen, 20.1 kg/ha 0.5 N NaHCO_3_ extractable phosphorus, 172.4 kg/ha 1.0 N NH_4_OAc exchangeable potassium, 8.2 pH and 0.72 dS/m electrical conductivity.

### Experimental details and crop management

The experiment was consisted of ten treatments (N management options) arranged in randomized block design in triplicates. Crop (var. Radhika-DRMR 2015–17) was sown in the second fort night of October during rabi season of 2021–22 and 2022–23 by following seed rate @ 3.5 kg/ha by adopting row to row and plant to plant distance of 45 x 10 cm. The intercultural operations including gap filling (4–6 DAS) and thinning was performed to maintain the optimum plant population. Recommended dose of fertilizers N, P_2_O_5_, K_2_O, S and B @ 80-40-40-40-1 kg/ha, respectively were applied to the crop by using urea, single super phosphate, murate of potash, sulphur in addition to borax. Initially, a basal application of nitrogen (50%) and PKSB (100%) was uniformly administered to all plots via soil, ensuring consistency across treatments. Subsequently, following the first irrigation (30–35 DAS), nitrogen was applied according to specific treatments, utilizing various methods tailored to meet the crop’s individual nutrient demands. This approach allowed for targeted nutrient management based on the crop’s evolving needs post-establishment, enhancing resource utilization and optimizing agricultural productivity. Thinning operation was carried out at 18–21 days after sowing to maintain optimum plant population by removing extra plants. Recommended agronomical management practices were adopted during crop growing season. Data on various parameters *viz*. plant height, branches primary as well as secondary/plant, siliquae (no.)/plant, siliquae length, seed (no.)/siliquae, 1000 seed weight, seed and stover yield were recorded. After reaching physiological maturity, mustard was harvested manually during month of March-April followed by drying and threshing and seed yield were recorded from all the treatment plots separately.

### GreenSeeker hand held crop sensor (GS)

GS is a cost effective, friendly, hand held measurement device, popularly known for assessing the healthiness or vigorness of any plant for making precise nutrient management. The device provide instant reading from plants about its health and these readings we used for making non-subjective decisions concerning quantity of nitrogen fertilizer need to be apply to the plants. The device gives more precise use of nitrogen to benefit the farmers as well as the ecosystem. The sensor through brief bursts of red and infrared light and measures their amounts which get reflected back by the green plant canopy. These measured values can be read from its LCD display screen which ranges from 0.00 to 0.99 and termed as NDVI (Normalized Difference Vegetation Index) readings. Observance or reflectance of incident light by the plant depends on the structure of the plant tissues, chlorophyll content. Chlorophyll contained in palisade layer absorb almost 70–90% of the all incident visible light (400–720 nm), whereas, the mesophyll tissues reflects about 60% of all incident NIR radiation (720–1300 nm) [15]. N-rich strips was prepared by following 150%, 200% & 300% (120, 160 & 240 kilograms of N per hectare) of nitrogen of RDF in split doses to ensure sufficiency of nitrogen. The NDVI was calculated with following expression:

$$NDVI=\frac{FNIR-FRed}{FNIR+FRed}$$

Where, F_NIR_ and F_Red_ are the fractions of emitted NIR and red radiation reflected back from the sensed area.

### Crop production efficiency (CPE)

The CPE of each nitrogen management options were obtained by dividing economic yield (seed) with its growing period in days (duration) [16]. The crop duration of 147 days during first year and 139 days during second year were taken for estimating the CPE using following equation:

Cropproductionefficiency(kg/ha/day)=Seedyield(kg/ha)/Cropduration(days)

The economic viability of every treatment was calculated on the basis of existing present market value of used input materials like seeds, fertilizers etc. during cultivation. The total cost of cultivation involved expenses of fuel for machinery, irrigation charges, man power, seed material, fertilizers and harvesting. The benefit cost ration was derived by using following formula [17].


$$Benefit-cost\;ratio\;(US\$/ha)=\frac{Gross\;monetary\;returns}{Total\;inputs}$$

### Statistical analysis

Recorded data on various parameters were subjugated to statistical analysis for assessing their significance using SAS 9.3 statistical software (IBM ver.23). Data on different parameters from two consecutive years taken as average to determine mean means and then subjected to ANOVA (analysis of variance) to define significance among the treatments. The treatment means were differentiated by DMRT [18] at *p =* 0.05 level of significance.

## Results

Data presented for both consecutive years in the respective tables or graphs. However, the interpretation of data and result writing was done on the mean data (mean of 2021–22 and 2022–23).

### Growth traits

Different N management options were affected growth attributes of *Brassica juncea* (L.) in both the consecutive years as well as on mean data basis. Growth traits *viz*. plant height (m), primary branches/plant did not affected significantly except secondary branches/plant. However, maximum plant height (2.07 m), primary & secondary branches/plant (6.8 and 14.0) were recorded when N was applied using GreenSeeker (GS) hand held crop sensor (Table 1), whereas values of these traits were observed minimum under control (1.9 m, 5.2 and 9.6), where no fertilizers were applied. Among the N foliar spray (NFS) treatments, application of 2% NCU was found superior over other NFS treatment and their levels (Urea and KNO_3_ @ 2.0 & 1.5% each) in improving plant growth parameters at both N levels (N_75_ & N_100_). Results obtained from N application through leaf colour chart (LCC) were at par with the results of GS.

**Table 1:** Effect of N management options on growth and yield parameters of mustard.

Treatments	Plant height (m)			Primary branches (no.)			Secondary branches (no.)			Siliquae/ plant (no.)			Siliquae length (cm)		
	2021–22	2022–23	Mean	2021–22	2022–23	Mean	2021–22	2022–23	Mean	2021–22	2022–23	Mean	2021–22	2022–23	Mean
T_1_	2.02	2.12	2.07	6.3	5.9	6.1	15.1	10.2	12.7	502	409	456	5.30	4.83	5.07
T_2_	2.02	2.15	2.09	6.5	6.0	6.3	15.8	10.3	13.1	516	413	465	5.33	4.87	5.10
T_3_	1.97	2.06	2.02	6.2	5.9	6.1	14.7	9.6	12.2	511	379	445	5.17	4.77	4.97
T_4_	1.96	2.07	2.02	6.1	5.9	6.0	15.0	9.3	12.2	483	381	432	5.17	4.67	4.92
T_5_	1.95	2.08	2.02	6.3	5.9	6.1	15.5	9.7	12.6	506	397	452	5.31	4.77	5.04
T_6_	1.96	2.06	2.01	6.1	5.7	5.9	14.0	8.9	11.5	474	369	422	5.11	4.67	4.89
T_7_	1.99	2.14	2.07	6.9	6.7	6.8	16.2	11.8	14.0	522	511	517	5.43	5.28	5.36
T_8_	1.99	2.12	2.06	6.2	6.5	6.4	16.1	11.6	13.9	486	483	485	5.20	5.03	5.12
T_9_	2.02	2.08	2.05	6.1	6.0	6.1	13.9	9.9	11.9	483	401	442	5.00	4.81	4.91
T_10_	1.81	1.99	1.90	4.9	5.5	5.2	10.6	8.5	9.6	370	369	370	4.88	4.59	4.74
LSD (p = 0.05)	0.12	NS	NS	NS	NS	NS	2.71	0.9	1.4	48.4	28	53.2	0.30	NS	NS

(T1: RDN100 (50% N basal + 50% N Top dressing) + 2% Urea FS; T2: RDN100 (50% N basal + 50% N Top dressing) + 2% NCU FS; T3: RDN100 (50% N basal + 50% N Top dressing) + 1.5% KNO3 FS; T4: RDN75 (50% N basal + 25% N Top dressing) + 2% Urea FS; T5: RDN75 (50% N basal + 25% N Top dressing) + 2% NCU FS; T6: RDN75 (50% N basal + 25% N Top dressing) + 1.5% KNO3 FS; T7: RDN–Green seeker; T8: RDN–LCC; T9: RDF; T10: Control)

### Yield attributes and yield

Data (Table 2) revealed significant enhancement with respect to yield traits (siliquae/plant, seed/pod, and test weight) and, yield (seed & stover) of *Brassica juncea* (L.) except siliquae length (Table 1) in the consecutive years (2021–22 & 2022–23). On mean basis, highest value (no.) of siliquae/plant, siliquae length, seed/pod and test weight recorded under GS sensor based N application, which were higher by 39.7, 13.0, 29.4 and 25.7 per cent over control treatment followed by N application based on LCC (31.0, 8.01, 25.7 and 25.6 per cent increment). Among foliar sprays of N made through different sources, 2% NCU was found superior over two other FS treatment (2% Urea and 1.5% KNO_3_) at N_100_ followed by N_75_. Improvement in yield attributes due to various N management options results in to higher seed and stover yield, harvest index which in turn to maximum net monetary benefit. The percent increase in seed yield was 19.3 and 64.5% higher under GS based N application in multi splits as compare to RDF and control followed by T_2_ (RDN_100_ (50% N basal-half dose + 50% N Top dressing-remaining half dose) + 2% NCU FS) and T_1_ (RDN_100_ (50% N basal-half dose + 50% N Top dressing-remaining half dose) + 2% Urea FS). Whereas, per cent yield enhancement in case of LCC based N application was 11.8 and 54.4 per cent, respectively over RDF and control. While, maximum stover yield was recorded under T_2_ and T_1_ in comparison to GS based N application. All N treatments gave better results of yield attributes and yield at N_100_ than N_75_. N management through GS and LCC gave higher values of harvest index (28.1 and 27.6) over rest of the treatments including control at lowest (24.3).

**Table 2:** Effect of N management options on growth and yield parameters of mustard.

Treatments	Seed/siliquae			1000 seed weight (g)			Seed yield (kg/ha)			Stover yield (kg/ha)			HI (%)		
	2021–22	2022–23	Mean	2021–22	2022–23	Mean	2021–22	2022–23	Mean	2021–22	2022–23	Mean	2021–22	2022–23	Mean
T_1_	17.7	16.1	16.9	6.12	5.31	5.72	2581	2693	2637	6774	8463	7619	27.6	24.1	25.9
T_2_	18.1	16.3	17.2	6.19	5.32	5.76	2653	2804	2729	6690	8604	7647	28.4	24.6	26.5
T_3_	17.7	15.8	16.8	5.74	5.22	5.48	2442	2673	2558	6596	8120	7358	27.0	24.8	25.9
T_4_	17.4	15.7	16.6	5.33	5.23	5.28	2424	2542	2483	6699	7934	7316	26.6	24.3	25.4
T_5_	17.5	16.1	16.8	6.00	5.18	5.59	2449	2644	2547	6201	8136	7169	28.3	24.5	26.4
T_6_	17.2	15.6	16.4	5.33	5.04	5.19	2395	2419	2407	6006	7763	6885	28.5	23.8	26.1
T_7_	18.3	16.9	17.6	6.28	5.34	5.81	2756	2962	2859	6254	8640	7447	30.6	25.5	28.1
T_8_	17.5	16.7	17.1	6.22	5.19	5.71	2490	2747	2619	5830	8103	6966	29.9	25.3	27.6
T_9_	17.1	16.1	16.6	5.22	5.03	5.13	2310	2377	2343	6413	7474	6943	26.5	24.1	25.3
T_10_	13.8	13.3	13.6	4.53	4.71	4.62	1676	1716	1696	4648	6008	5328	26.3	22.2	24.3
LSD (p = 0.05)	1.60	0.89	0.92	0.12	0.18	0.14	221	465	329	1099	1234	1158	NS	NS	NS

(T1: RDN100 (50% N basal + 50% N Top dressing) + 2% Urea FS; T2: RDN100 (50% N basal + 50% N Top dressing) + 2% NCU FS; T3: RDN100 (50% N basal + 50% N Top dressing) + 1.5% KNO3 FS; T4: RDN75 (50% N basal + 25% N Top dressing) + 2% Urea FS; T5: RDN75 (50% N basal + 25% N Top dressing) + 2% NCU FS; T6: RDN75 (50% N basal + 25% N Top dressing) + 1.5% KNO3 FS; T7: RDN–Green seeker; T8: RDN–LCC; T9: RDF; T10: Control)

### Profitability

**Fig 1:**
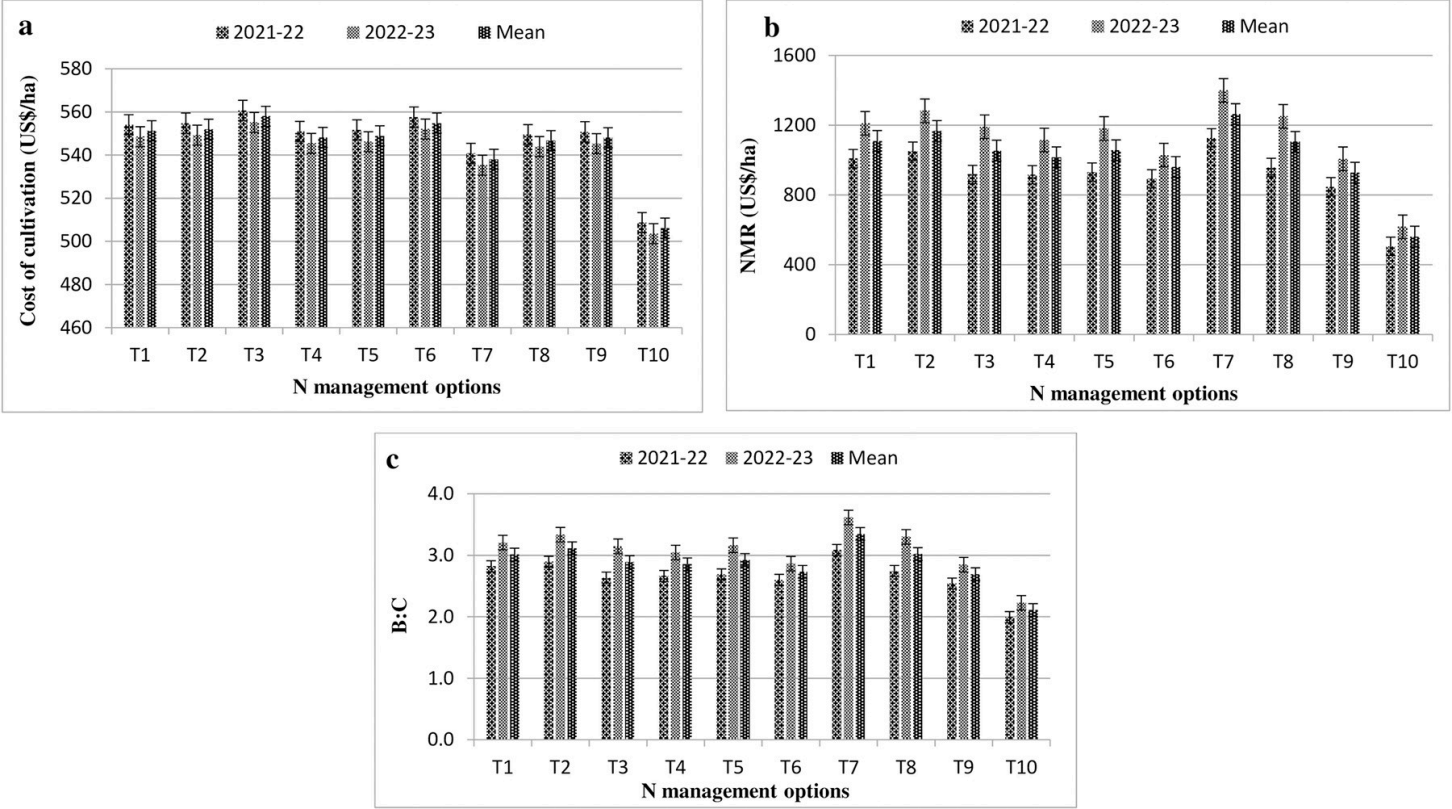
Effect of N management options on profitability of different treatments in mustard a) cost of cultivation; b) NMR; c) B:C. (T_1_: RDN100 (50% N basal + 50% N Top dressing) + 2% Urea FS; T_2_: RDN100 (50% N basal + 50% N Top dressing) + 2% NCU FS; T_3_: RDN100 (50% N basal + 50% N Top dressing) + 1.5% KNO_3_ FS; T_4_: RDN75 (50% N basal + 25% N Top dressing) + 2% Urea FS; T_5_: RDN75 (50% N basal + 25% N Top dressing) + 2% NCU FS; T_6_: RDN75 (50% N basal + 25% N Top dressing) + 1.5% KNO_3_ FS; T_7_: RDN—Green seeker; T_8_: RDN—LCC; T_9_: RDF; T_10_: Control).

Adoption of various N management options were significantly affected economics as well as viability of the different treatments (*viz*. cost of production, net monetary return-NMR, benefit-cost ratio etc.). The minimum expenditure with respect to cost of production/cultivation was incurred in GS based split N application (US$ 538/ha) in comparison to other treatments except control (no fertilizer application) (Fig 1A). Under NFS treatments, N application through KNO_3_ at both N levels (N_75_ & N_100_) accomplished higher cost of production. With respect to NMR and BC ratio, N application through GS hand held crop sensor fetched maximum values followed by T_2_ (N_100_ + 2% FS-NCU) and T_8_ (LCC) treatment. The per cent increase in NMR and BC ratio was 125.1 & 36.2% and 58.8 & 24.4%, respectively over control and RDF under GS based N application (Fig 1B & 1C) while control fetched least values of both these parameters (US$ 561/ha & 2.11) among all the treatments.

### Cost per kilogram and crop production efficiency (CPE)

**Fig 2:**
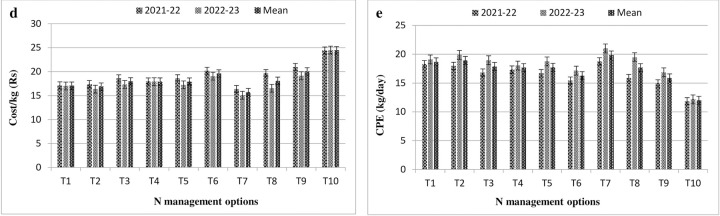
Effect of N management options on cost per kilogram (Fig d) and crop production efficiency (Fig e) in mustard. (T_1_: RDN100 (50% N basal + 50% N Top dressing) + 2% Urea FS; T_2_: RDN100 (50% N basal + 50% N Top dressing) + 2% NCU FS; T_3_: RDN100 (50% N basal + 50% N Top dressing) + 1.5% KNO_3_ FS; T_4_: RDN75 (50% N basal + 25% N Top dressing) + 2% Urea FS; T_5_: RDN75 (50% N basal + 25% N Top dressing) + 2% NCU FS; T_6_: RDN75 (50% N basal + 25% N Top dressing) + 1.5% KNO_3_ FS; T_7_: RDN—Green seeker; T_8_: RDN—LCC; T_9_: RDF; T_10_: Control). * Data on cost/kg was not expressed in USD due to smaller values.

In both the years, data indicated significant changes in the cost of production and their production efficiency on applying various N application treatments. Among the treatments, lowest production cost per kilogram (Rs 15.7/kg) was obtained in the treatment when N applied with the help of GreenSeeker hand held crop sensor in multiple splits (Fig 2D & 2E) followed by treatment T_2_ and T_1_ at par with each other. In foliar sprays of N, 1.5% KNO_3_ required more cost for producing one kilogram of seed due to getting of lesser yield in comparison, followed by RDF and LCC too. The production cost was highest in control (Rs 24.45/kg) as compare to all other treatments. Data further exhibited that N management through GS acquired higher values of CPE (19.9 kg/day) closely followed by FS treatments and LCC over the control (12.0 kg/day). The value of CPE varies from 15.9 to 19.9 kg/day among the different N management options.

### Oil content and oil yield

**Fig 3:**
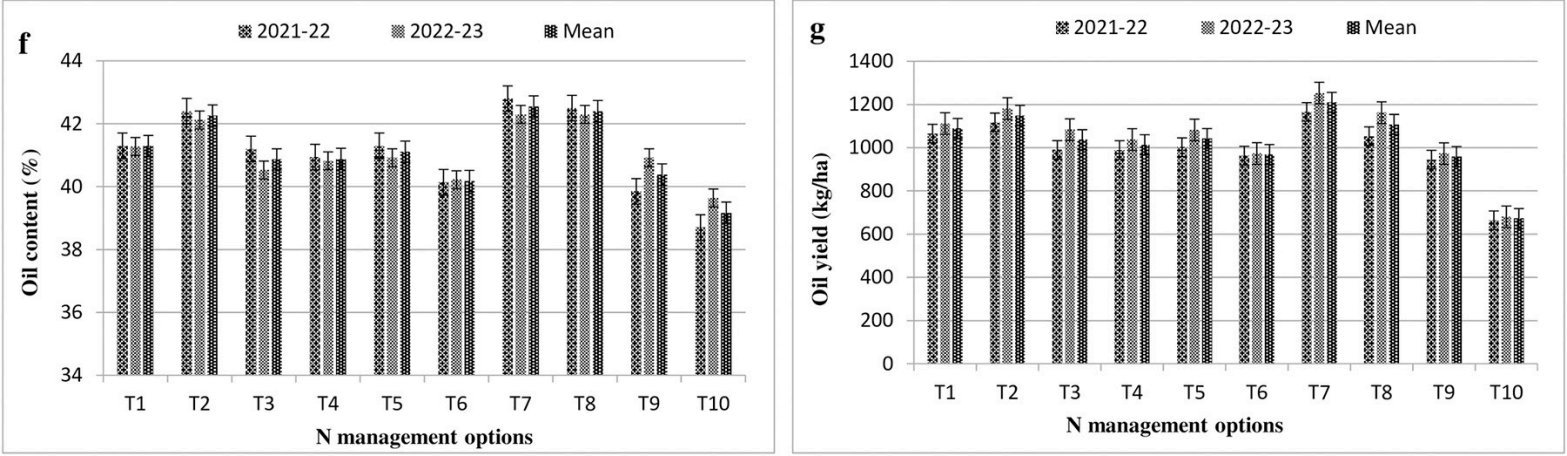
Effect of N management options on oil content (Fig f) and oil yield (Fig g) in mustard. (T_1_: RDN100 (50% N basal + 50% N Top dressing) + 2% Urea FS; T_2_: RDN100 (50% N basal + 50% N Top dressing) + 2% NCU FS; T_3_: RDN100 (50% N basal + 50% N Top dressing) + 1.5% KNO_3_ FS; T_4_: RDN75 (50% N basal + 25% N Top dressing) + 2% Urea FS; T_5_: RDN75 (50% N basal + 25% N Top dressing) + 2% NCU FS; T_6_: RDN75 (50% N basal + 25% N Top dressing) + 1.5% KNO_3_ FS; T_7_: RDN—Green seeker; T_8_: RDN—LCC; T_9_: RDF; T_10_: Control).

Applying various N treatments significantly enhanced both oil per cent and oil yield of *Brassica juncea* (L.) owing to their application method effect during both cropping years and on mean basis. Maximum mean oil content (42.3%) was achieved by application of N through GS and LCC methods each, closely followed by FS of 2% NCU at N_100_ level (42.1%), while remaining treatments were at par to each other (Fig 3F & 3G). Further, the minimum per cent of oil was obtained in control treatment (39.6.), where no fertilizers were applied. Similar trends of results were also observed with respect to oil yield in both the years. Application of N with GS registered for higher oil yield over other treatment means. The per cent increment in oil yield owing to GS based nitrogen application was estimated about 79.9% above control and by 26% higher over RDF. Whereas, N management through FS by various sources were more or less equal to each other at both the N levels (N_75_ & N_100_) except treatment T_2_ (FS 2% NCU). Due to lesser seed yield and oil content, control treatment registered with lowest oil yield (672 kg/ha).

### SPAD and NDVI

**Fig 4:**
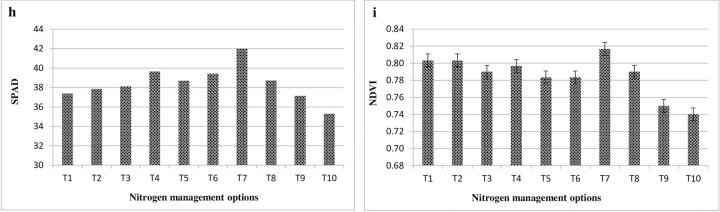
Effect of N management options on SPAD (Fig h) and NDVI (Fig i) in mustard (Mean data of two years). (T_1_: RDN100 (50% N basal + 50% N Top dressing) + 2% Urea FS; T_2_: RDN100 (50% N basal + 50% N Top dressing) + 2% NCU FS; T_3_: RDN100 (50% N basal + 50% N Top dressing) + 1.5% KNO_3_ FS; T_4_: RDN75 (50% N basal + 25% N Top dressing) + 2% Urea FS; T_5_: RDN75 (50% N basal + 25% N Top dressing) + 2% NCU FS; T_6_: RDN75 (50% N basal + 25% N Top dressing) + 1.5% KNO_3_ FS; T_7_: RDN—Green seeker; T_8_: RDN—LCC; T_9_: RDF; T_10_: Control).

SPAD and NDVI readings were taken from the plants under all treatments. The sensor based instruments used for SPAD readings was SPAD-502Plus (Konica Minolta) and NDVI (Normalized difference vegetation index) was measured with the help of Green Seeker hand held crop sensor (Trimble Agriculture). Data on SPAD value (**Fig 4H**) revealed that GS based nitrogen application recorded higher value (42.0) on mean basis which is the indication of leaf chlorophyll content that showed higher readings means high chlorophyll content due to more leafy greenness because of consistent availability of nitrogen to the plants and makes plant healthier and more vigours. The ranges of SPAD reading was lies in between 35.3 (control-minimum) to 42.0 (GS-maximum). Alike SPAD meter, the NDVI values was measured higher under N application when applied precisely using Green Seeker crop sensor (0.82) as compare to control (0.74) (**Fig 4I**). Higher values of NDVI represents the more leaf chlorophyll content i.e. leaf N concentration. The response of foliar N application at pre-flowering stage results better in terms of more flower and pod development which ultimately turns into to higher seed yield. Foliar application of N @ 2% FS through neem coated urea and urea both are equally effective (0.80) as compare to their lower N levels (1.5% FS) while FS of KNO_3_ was not as much as effective. The readings of SPAD and NDVI higher or lower depend on the leaf greenness due to N application which is the direct indicator of leaf N content.

**Fig 5:**
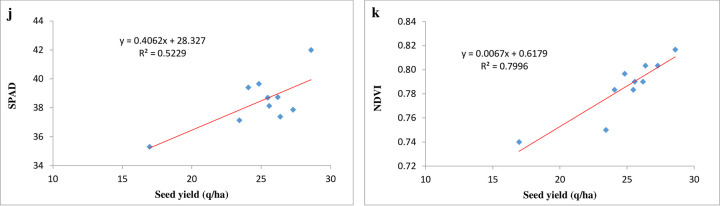
Linear regression line exhibiting relationship between j) seed yield and SPAD, k) seed yield and NDVI.

The regression analysis between mustard seed yield and SPAD value exhibited significantly positive linear relationship (**Fig 5J**). The seed yield was positively correlated with SPAD value (r^2^ = 0.52). The relationship explained that seed yield had 52.29 per cent variability of the SPAD value. Similarly, NDVI has positive association with the mustard seed yield showing 79.96 (r^2^ = 0.80) per cent variability (**Fig 5K**). The higher values of SPAD or NDVI was due to more leaf N content because of frequent supply of N to the plant from soil available nitrogen.

### Vicissitudes in percent SOC and yield with N management options

Significant changes were observed pertaining to SOC (soil organic carbon) content from its initial values after experiments for two consecutive years under in all the treatments. Under N treatments, the decline in SOC content was greater in foliar spray of N with -1.5% KNO_3_ (23% & 16.7% reduction) at both N levels (N_75_ and N_100_), whereas, in all other treatments, there were positive per cent increase in SOC content from its initial values (Table 3) ranges from 3.7% to 18.7%, respectively. The per cent increase in SOC content under GS based N application was 16.1% closely followed by LCC (15.6), while RDF recorded least positive changes in SOC (3.7%) from its initial value. However, highest percent reduction in SOC content (-22%) was observed under control, where no fertilizers applied. Similarly, percent N saving was greater under GS guided N management option (18.7%) due to precise N application over RDF (80 kg N/ha). Nitrogen foliar spray treatments at N_100_ level (T_1_, T_2_ & T_3_) registered for negative percent changes (-1.46 to -6.90%) in N over RDF due to excess N application in the form of FS. However, maximum percent N saving was recorded under T_6_ (NFS @ 1.5% KNO_3_) due to partial N application at N_75_ (61.17 kg N/ha) over RDF. The percent changes (increase) in the seed yield of mustard were varied from 3.70% (minimum- T_6_) to 19.31% (maximum- T_7_). However, highest percent seed yield decline was recorded in the control treatment (-27.46%).

**Table 3:** Effect of N management options on organic carbon (%), nitrogen and yield of mustard.

Treatments	Initial OC (%) before expt.	OC (%) after two years expt.	% change in OC	N Applied	% N saving over RDF	% Change in SY over RDF
T_1_	0.38	0.45	18.7	85.52	-6.90	11.74
T_2_	0.42	0.47	10.8	85.52	-6.90	14.84
T_3_	0.44	0.37	-16.7	81.17	-1.46	5.73
T_4_	0.39	0.42	9.6	65.52	18.10	4.92
T_5_	0.40	0.45	13.5	65.52	18.10	6.04
T_6_	0.44	0.34	-23.0	61.17	23.54	3.70
T_7_	0.41	0.48	16.1	65	18.75	19.31
T_8_	0.40	0.47	15.6	72	10.00	7.81
T_9_	0.42	0.44	3.7	80	-	-
T_10_	0.41	0.31	-22.5	-	-	-27.46
LSD (p = 0.05)	NS	0.06	-	-	-	-

(T1: RDN100 (50% N basal + 50% N Top dressing) + 2% Urea FS; T2: RDN100 (50% N basal + 50% N Top dressing) + 2% NCU FS; T3: RDN100 (50% N basal + 50% N Top dressing) + 1.5% KNO3 FS; T4: RDN75 (50% N basal + 25% N Top dressing) + 2% Urea FS; T5: RDN75 (50% N basal + 25% N Top dressing) + 2% NCU FS; T6: RDN75 (50% N basal + 25% N Top dressing) + 1.5% KNO3 FS; T7: RDN–Green seeker; T8: RDN–LCC; T9: RDF; T10: Control)

## Discussion

### Growth attributes

Due to involvement of nitrogenous fertilizers into various growth and developmental processes of plants, they became more indispensable for sustainable food production. Simultaneously their uses in excess quantities beyond the recommended level are most commonly adopted tactics though out the globe for meeting nutrients requirement of the plants. Further, poor nitrogen management attributed to innumerable nitrogen losses via leaching, denitrification and volatilization in straight fertilizers [19], causing low nitrogen recovery (rarely exceeds 30–35%) in soil profile. Further, inadequate soil nitrogen supply resulted into deceased dry biomass production in succeeding plant growth phases. These losses can be minimized to least as possible by adopting innovative agronomic management tactics leads to augmented yield. Coating of nitrogen fertilizers decelerated the rate of ammonia release and synchronizes N supply with the plant requirement as and when needed without any interruption in N supply throughout the growing period, thereby increasing plant capacity to harvest more dry biomass and economic yield [20]. In this direction, urea coating is a simple and cost effective approach. Further, root growth and nutrient acquisition in the plants could be augmented by the use of slow nutrient releasing fertilizers [21]. Quick evaluation of nitrogen concentration in leaves is a sensitive indicator linked to plant nitrogen necessity during growing period leads to the dynamic N fertilizer management. LCC is another such diagnostic tool that monitors relative leaf greenness rapidly and reliably used for knowing the real status of leaf N content.

### Yield and yield attributes

Application of different N management options significantly improved all the growth attributes such as plant height, branches/plant (primary & secondary), number of siliquae and length, leaf greenness (SPAD values) etc. which increases plant biomass. GreenSeeker based N application recorded maximum plant height (8.94% more), primary and secondary branches per plant (30.8 & 45.8% higher), siliquae per plant (39.7% more), siliquae length (13.0% higher), respectively over control on mean basis. This could be due to increased soil N availability that augmented the uptake of nutrients by crop. Further, synchronization in plant nitrogen demand and soil N supply reduces wastage of nitrogen fertilizers. Similar results were also revealed by others [22, 23].

The yield is fraction of total dry biomass produced in the form of seed governing by several bio-physiological processes [24] which indicate strong relationship between the source-sink. Application of nitrogen in two splits– 50% as basal and 50% at first irrigation (35–40 DAS) is a general recommendation, found effective for improving seed yield and NUE in mustard. A significant improvement in *Brassica juncea* (L.) seed harvest and dry matter production was registered owing to application of N by using GS hand held crop sensor as compare to other N management options. The average data of two year indicated that GS based N application in multi splits increased yield significantly by 19.3 and 64.5 per cent over RDF and control treatment. Due to enhanced soil nutrient availability and harmonization in crop N requirement and soil N supply through split application improves soil fertility status and created congenial environment to the plant growth. Further, due to increased photosynthetic partitioning towards vegetative and reproductive parts occur concurrently at later growth stages [25]. This may ascribe in overall improvements in the vigour and growth of mustard. Moreover, the augmentation in seed yield may be brought due to values of yield parameters, viz. branches/plant (6.8 primary & 14.0 secondary branches), siliquae/plant (517), seeds/siliquae (17.6) and test weight (5.81 g). The improvement in yield traits is the outcome of sufficiency of nutrients to plants as per the need which boosted growth and translocate more assimilates toward sink from source [26]. These results are corroborated with the findings of Chaudhary et al. [27].

Moreover, N application in multiple splits lessens the losses of N arose due to various means like volatilization, leaching, denitrification etc. as earlier studies showed that about 50% of N lost to the environment by these losses. Reduction in N losses ultimately benefitted the crop as maximum N is taken up by the plant to improve their growth and development. The findings reported by Singh et al. [28] also suggested that GS crop sensor gave better results in irrigated wheat by managing nitrogen efficiently in the region of “Indo-Gangetic plains of South Asia” and concluded that optical sensor based N application might be the paramount approach for achieving higher NUE. The principal behind this practice is feeding the growing plants instead of feeding soil by harmonizing plant nutrient demand with supply. Assessing plant nutrient requirement is one of the more effective approaches as “plant growth assimilates influence of nutrients supplied from all accessible sources” at given time and therefore it considered as a credible indicator of their availability. These may be attributed to maximum crop growth in case of precision nutrient management [29]. N application when crop requirement is higher diminishes occurrence of N losses via soil-plant system and eventually benefitted the plants with enhanced nitrogen use efficiency. Similar findings was also revealed by Jiang et al. [30] indicated that nitrogen management in potato using Nutrient Expert (NE) significantly augmented yield by 7 per cent with 21 per cent reduced nitrogen and declined the N losses and N balance by 31 and 48 per cent, respectively. Further, use of Nutrient Expert decreased N requirement by 19 kg/ha with no yield reduction and/or profit loss over the soil test and minimized losses as well as balance of nitrogen by 13 and 27 per cent, respectively. Earlier study is also reported that applying lesser quantity of N converted more efficiently into higher yield as compare to higher doses [31]. The present findings were supported by previous literatures indicated that yield traits were significantly improved while using NE and green seeker for N management [29, 32].

The demand based variable-rate fertilizer application is another important tactic for accomplishing better crop growth and development, improved yield traits that eventually come out with maximum economic product and revamped nutrient acquisition by solving the problematic situations of excess or lesser fertilization [33]. A positive response between yield and split N application may be attributed to higher leaf nitrogen content that enhanced and prolonged photosynthetic activity of plants that could be responsible for attaining higher yield [34, 35]. During initial stage, nitrogen requirement of plant can be fulfill by the native soil N to complete its vegetative growth in sufficient quantity and also seed itself has enough food to complete its germination and further processes at seedling stage. The greater influence of soil N availability on growth and yield was seen as soon as peak N demand of the crops arises during later phases. Meena et al. [36] who also confirmed splitting of nitrogen at later growth phases augmented maize grain and total dry biomass yield by 16.3% & 11.2% and AE, PFP, PE and RE by 45.1%, 15.3%, 14.0% & 37.9% as compared to conventional N fertilizer recommendation. Further, it also supported by the fact that during initial stage (germination and seedling stage), application of half of the nitrogen (about 50% of recommended) as basal might have lost due to leaching, denitrification, volatilization, ecosystem saturation and polluting water bodies as at this stage plant seedlings shows incapability to utilize the applied higher N doses due to poor root system [37]. Earlier literatures also showed augmented content of soil ammonia levels due to more application of N as basal during early vegetative growth causes enormous N losses [38–40]. Additionally, foliar spray of N fertilizers either sole or in combination increased yield in many crops at critical stages because of direct penetration of nutrients to the plant tissues via stomata holes which further transported through plasmodesmata [1]. Amongst foliar sprays of N, FS of 2% NCU was found superior over other FS treatment (2% Urea and 1.5% KNO_3_). Studies revealed that *Azadirachta indica* nitrification inhibitors reduced the losses of nitrogen and higher content of nitrate as in case of commercially used urea [41]. The findings were corroborated with results reported by Gangurde et al. [42] and Geng et al. [43], mentioned that coating of nitrogen fertilizers increased soil N availability (188.40 kg ha^−1^).

### Profitability

Applying more quantities of inputs with other field operations followed in traditional practices cumulatively raises cost of production while reducing net profitability [24]. The incremental rate of net returns was lesser under control (US$ 561/ha) followed by RDF (US$ 927/ha), indicating non-responsiveness as well as non-economical for augmenting *Brassica juncea* production. The cost of cultivation (US$/ha) was registered higher under nitrogen FS treatments (2.02–3.70% higher) over GS based N management owing to higher cumulative cost of fertilizers and labour charges, whereas, the minimum production cost was recorded under control, where no fertilizer added. Adoption of N through GS increased the net monetary benefit by 36.2% and 125.1% over RDF and control. Similar outcomes were also found by earlier investigators [28, 30].

### Oil content and oil yield

The oil percent in seed exhibited inverse correlation with different levels of N and hence, oil percent was decreased with raising doses of nitrogen. Nitrogen management through GS and LCC both registered for higher oil content (42.3% each) which were at par with treatment T_2_ (42.1) as compare to RDF (40.9%). The mustard seed oil percent was decreased due to increase of protein substance (proportion) because of augmented soil N availability [44]. In case of applying more quantity of N, diversion of photosynthates to protein formation may be seen in huge proportion which causes potential deficit of carbohydrates to be degraded to “acetyl co-enzyme A” required for formation of fatty acids. Similarly, oil yield is a simple multiplication of seed yield and oil percent. Getting higher oil content and seed harvest was ascribed for maximum oil yield (T_7_-1209 kg/ha). The per cent increase in oil yield varied from 0.93 to 26.1% over the RDF, while control treatment achieved lesser values (29.9% less). Additionally, increase in oil percent with oil yield could be ascribed to occurrence of fatty acids synthesis in plants by transformation of acetyl coenzyme A into malonyl coenzyme A in the presence of Adenosine triphosphate plus phosphate. Similar findings were also reported by other investigators [45].

## Conclusion

Nitrogen is subjected to heavy losses in the form of leaching, volatilization, or denitrification and an abundance of N in soil at seedling stage might have occasionally result in significant losses of N fertilizers due to occurrence of heavy rainfall. Experimental data exhibited that GS optical sensor based N management reduces N requirement by 15–20%, while simultaneously maintaining sustainable yield levels. In comparison to RDF/farmers practices, use of GreenSeeker optical sensor devise optimized total N inputs, and synchronized crop N requirement with soil N supply. To provide enough N availability throughout the crop growing season, application of N in multi splits are pertinent rather than top dressing the remaining 50% N at the initial irrigation which results into improved productivity, profitability and sustainability. This approach may also help in reducing the occurrences of N_2_O production by decreasing availability of mineral N for microbial activities.
